# The psychological impacts of COVID-19: a study of frontline physicians and nurses in the Arab world

**DOI:** 10.1017/ipm.2020.119

**Published:** 2020-10-28

**Authors:** N. Al Mahyijari, A. Badahdah, F. Khamis

**Affiliations:** 1 The Royal Hospital, Ministry of Health, P.O. Box 1331 PC111, Muscat, Oman; 2 Department of Sociology and Rural Studies, South Dakota State University, Hansen Hall 004, Brookings, SD 57007-2201, USA

**Keywords:** Anxiety, COVID-19, Oman, Stress, Well-being

## Abstract

**Objectives:**

The COVID-19 (SARS-CoV2) pandemic is wreaking havoc on healthcare systems and causing serious economic, social, and psychological anguish around the globe. Healthcare workers (HCWs) who diagnose and care for COVID-19 patients have been shown to suffer burnout, stress, and anxiety.

**Methods:**

In this study, we collected data from 150 frontline HCWs who had close contact with COVID-19 patients at several health facilities in the Sultanate of Oman. The participants completed an online survey that included the Perceived Stress Scale, the Generalized Anxiety Disorder Scale, and the WHO-5 Well-Being Index.

**Results:**

The study found that a substantial number of healthcare professionals experienced relatively high levels of stress and anxiety, as well as suboptimal levels of well-being. Perceived stress and anxiety were significant predictors of HCWs’ well-being.

**Conclusions:**

This study adds to the increasing literature indicating harmful effects of COVID-19 on the mental health of HCWs.

## Introduction

On January 30, 2020, the World Health Organization (WHO) declared SARS-CoV-2 outbreak a Public Health Emergency of International Concern and on February 11, 2020, it named the disease caused by the new virus COVID-19. On March 11, the WHO declared the outbreak a pandemic (WHO, 2020a). Since then, the COVID-19 (SARS-CoV2) pandemic has posed a serious threat to humanity. At the beginning of the outbreak, there were around 75 000 confirmed cases in China (Liang *et al.*, 2020). As of September 13, 2020, there were about 28 584 158 confirmed positive cases and 916 955 deaths worldwide and about 88 337 confirmed cases and 762 deaths in Oman (WHO, 2020b). With a rapid increase in the number of infections and deaths and no vaccine or treatment on the horizon, the COVID-19 pandemic is creating extreme stress for healthcare systems around the globe (Druss, 2020). Both the public and healthcare workers (HCWs) are facing a host of social and psychological problems, including isolation, loneliness, stress, and anxiety. In a recent national survey by the Kaiser Family Foundation, 56% of American adults reported being worried or stressed because of COVID-19 (Kaiser Family Foundation 2020).

HCWs around the world put themselves in harm’s way to diagnose, treat, and care for COVID-19 patients, sometimes with limited protective personal equipment (PPE). Although there is no precise data on the number of HCWs who have been infected with COVID-19, WHO (2020c) data indicates that as of April 8, 2020, there were 22 073 infected HCWs in 52 countries. In the United States of America, as of April 14, 2020, there were almost 10 000 cases among HCWs (CDC, 2020). During this pandemic, HCWs are working in a continually stressful, challenging, and changing environment (Shanafelt *et al.*, 2020). Thus, fighting the COVID-19 pandemic and supporting and protecting frontline HCWs who care for its victims are two sides of the same coin.

Signs of mental health problems among HCWs during disease outbreaks have been observed in many healthcare settings (Lai *et al.*, 2020). Liang *et al.* (2020) reported symptoms of depression and anxiety among doctors and nurses in China during the earlier months of the COVID-19 outbreak. Similarly, Zhang *et al.* (2020) found higher rates of insomnia, anxiety, depression, and obsessive-compulsive symptoms among HCWs compared to non-medical HCWs.

In the long term, HCWs might experience harsher and different types of psychological morbidities, as observed during previous disease outbreaks such as SARS. For example, Lee and colleagues (2007) found 1 year after the outbreak of SARS that HCWs had higher levels of post-traumatic stress, depression, and anxiety compared to non-HCWs.

Research on the impact of COVID-19 on the mental health of HCWs in the Arab world is sparse. To amend this gap, the present study sought to investigate the impact that the COVID-19 outbreak has had on the mental health of HCWs who care for COVID-19 patients in Oman. Learning about the impact of this global pandemic on HCWs would help in developing administrative, psychological, and social supports for HCWs in Oman.

## Methods

The participants in this study are a subset of a larger study on COVID-19 that we conducted in Oman which included physicians, nurses, and non-medical personnel, including allied health professionals, laboratory technicians, housekeepers, and administrators. The analyses presented here pertain to physicians and nurses who provided care for COVID-19 patients.

### Participants

The sample consisted of 150 nurses (60.7%, *n* = 91) and physicians (39.3%, *n* = 59) recruited from several health facilities in Oman via emails and text messages. Half of the physicians (50.1%, *n* = 30) and most of the nurses were females (74.1%, *n* = 86). The participants completed an online survey that contained three psychological assessment tools and several demographic questions.

### Measures

To gauge the mental health of frontline clinicians, three widely used measures were employed: the Perceived Stress Scale (Cohen & Williams, 1988), the Generalized Anxiety Disorder Scale-7 (GAD-7) (Spitzer *et al.*, 2006), and the WHO-5 Well-Being Index (Topp *et al.*, 2015). We also collected information about age, gender, marital status, and number of years in practice.

#### Perceived stress scale-10 (PSS-10)

This is a well-established 10-item self-report that assesses stress management and whether events are perceived as stressful (Taylor 2015). Four items are positively worded (e.g. ‘In the last month, how often have you felt that things were going your way?’) and six are negatively worded (e.g. ‘In the last month, how often have you been upset because of something that happened unexpectedly?’). Participants are asked to assess their feelings and thoughts during the past month using five response levels ranging from 0 = *Never* to 4 = *Very often*. Total possible scores range from 0 to 40. A high score signifies a high level of stress. The PSS-10 has no predefined cutoff values. The Cronbach’s alpha coefficient for the PSS-10 in this study was 0.81.

#### Generalized anxiety disorder-7 scale (GAD-7)

This is one of the most used scales to gauge anxiety in both research and clinical settings (Toussaint et al., 2020). The GAD-7 is a self-report screening tool. which consists of seven items that ask participants about symptoms they have experienced in the past 2 weeks (e.g. ‘Feeling nervous, anxious, or on edge’). All items are rated on a 4-point Likert scale ranging from 0 = *Not at all* to 3 = *Nearly every day*, with total scores ranging from 0 to 21. High scores are indicative of greater anxiety. The total scores can be divided into four levels of anxiety: minimal (0–4), mild (5–9), moderate (10–14), and severe (15–21). A score of 10 or higher signifies a higher level of anxiety. In this study, the Cronbach’s alpha coefficient for the GAD-7 was 0.85.

#### WHO five well-being index (WHO-5)

The WHO-5 is a subjective short scale developed by the WHO in 1998 to measure current mental well-being (Topp *et al.*, 2015). It has also been successfully used to screen for depression (Krieger *et al.*, 2014). It consists of five items that require participants to rate their state of well-being during the preceding 2 weeks (e.g. ‘Over the last 2 weeks I have felt cheerful and in good spirits’). All items are rated on a 6-point Likert scale ranging from 0 = *None of the time* to 5 = *All the time*. The total score ranges from 0 to 25. In practice, however, the raw scores are multiplied by 4 to transform them into a percentage value. This procedure creates new scores that range from 0 to 100, with 100 being the optimal level of well-being. In the present study, the Cronbach’s alpha coefficient for the WHO-5 was 0.88.

## Results

The age of the participants ranged from 24 to 63 (*M*_age_ = 37.62, SD = 7.79). The majority were married (78.7%), followed by singles (16%) and others (5.3%). As shown in Table 1, most of the participants (77.3%) were females. Of this sample, 39.3% were physicians and 61% were nurses. On average, the HCWs had been in practice for 13.78 years (SD = 7.97). Physicians were slightly older (*M*_age_ = 40.28, SD = 9.73) than nurses (*M*_age_ = 35.83, SD = 5.57).

**Table 1.** Sociodemographic characteristics of participants

	Occupation				
	Physicians		Nurses		Full sample	
Characteristics	*n*	%	*n*	%	*n*	%
Gender						
Females	30	58.8	86	94.5	116	77.3
Males	29	49.2	5	5.5	34	22.7
Marital status						
Married	47	79.7	71	78	118	78.7
Unmarried^a^	12	20.3	20	22	32	21.3
Age						
24–36	18	41.9	37	57.8	55	51.4
37–63	15	58.1	27	42.2	52	48.6

N = 150. The total might not tally because of missing data.

a The unmarried category includes never married, divorced, and widowed.

As shown in Table 2, the mean score on the PSS-10 was 23.61 (SD = 6.47), with the lowest score being 1 and the highest 34. An independent-samples *t*-test examining the influence of gender on the PSS-10 was not significant (*t* = 0.35, *p* = .73). Physicians and nurses experienced comparable levels of stress (*t* = 0.23, *p* = .82). Older HCWs reported less stress (*r* = −.30, *p* = .002).

**Table 2.** Gender and occupation differences in stress, anxiety, and well-being

Variable	Characteristics	*t*/*χ*^*2*^	*p-*value
PSS-10	Male (23.26 ± 6.27) Female (23.71 ± 6.55)	.35	.73
	Physician (23.46 ± 6.94) Nurse (23.70 ± 6.18)	.23	.82
GAD-7	Male 5 (11.6%) scored ≥10 Female 38 (88.4%) scored ≥10	3.88	.04
	Physician 18 (41.9%) scored ≥10 Nurse 25 (58.1%) scored ≥10	.22	.39
WHO-5	Male 13 (16.7%) scored ≤ 50 Female 65 (83.3%) scored ≤ 50	2.87	.07
	Physician 32 (41%) scored ≤ 50 Nurse 46 (59%) scored ≤ 50	.21	.39

GAD-7, Generalized Anxiety Disorder-7; PSS-10, Perceived Stress Scale-10; WHO-5, WHO five Well-Being Index.

The HCWs’ score on the GAD-7 ranged from 0 to 20 (M = 7.43, SD = 4.64). Significantly more females had moderate or severe anxiety compared with males (Table 2). As shown in Table 2, no significant difference was found between physicians and nurses (*χ*^*2*^ (1, *N* = 149) = .22, *p* = .39). Age was not related to GAD-7 scores (*r* = −.09, *p* = .32).

WHO-5 scores ranged from 4 to 96 (M = 50.54, SD = 22.57). Conventionally, scores of ≤ 50 indicate poor psychological well-being that warrants screening for depression (Topp *et al.*, 2015). Slightly more than half (53.8%) of HCWs scored ≤ 50. As shown in Table 2, no significant differences were observed between males and females (*χ*^2^ (1, *N* = 149) = 2.87, *p* = .07) or between physicians and nurses (*χ*^2^ (1, *N* = 145) = .21, *p* = .39). Scores on the WHO-5 were not correlated with age (*r* = .14, *p* = .15).

Not unexpectedly, all three variables in the study were highly correlated, as shown in Table 3. There was a significant strong positive relationship between the PSS-10 and the GAD-7 (*r* = .40, *p* < 0.001). We also found significant strong negative relations between the PSS-10 and WHO-5 (*r* = −.46, *p* < 0.001) and the GAD-7 and WHO-5 (*r* = −.56, *p* < 0.001). We performed a multiple regression analysis in which PSS-10 (perceived stress) and GAD-7 (generalized anxiety) scores were entered as predictors of WHO-5 (well-being) scores. The model explained 61% of the variance (*R*^2^ = .37) in reported well-being scores (*F* (2, 142) = 41.62, *P* < 0.001). Both the PSS-10 (*β=* −.27*, t* (144) = 3.64, *p* < 0.001) and the GAD-7 (*β =* −.44*, t* (144) = 5.96, *p* < 0.001) significantly predicted WHO-5 Well-Being Index scores.

**Table 3.** Correlations and descriptive statistics for all variables in the study

	(1) PSS-10 correlation	(2) GAD-7 correlation	*M*	*SD*
(1) PSS-10			23.61	6.47
(2) GAD-7	.40**		7.43	4.64
(3) WHO-5	-.46**	-.56**	50.54	22.57

**Correlation significant at the <0.001 level (two-tailed).

## Discussion

This study investigated the mental health of HCWs who cared for patients with COVID-19 in Oman. The stress experienced by physicians and nurses is surprisingly high compared to the level reported in previous health- and non-health-related studies (e.g. Cohen & Janicki-Deverts 2012; Nielsen *et al.*, 2008; Nordin & Nordin, 2013). The mean score of 24 on the PSS-10 observed in the current study was higher than the mean of 15.97 reported during the lockdown in Austria (Pieh *et al.*, 2020) and the mean of 17.41 obtained from 41 countries during COVID-19 (Limcaoco *et al.*, 2020). We also found that older HCWs were less likely to experience stress compared to younger ones. We speculate that older HCWs have more experience and perhaps have dealt with other health crises during their career.

Another important finding associated with stress was the number of participants who experienced anxiety. Almost one-third of the HCWs reported moderate to severe anxiety, with no significant difference between physicians and nurses. Some previous studies have reported a lower percentage of participants with moderate to severe anxiety compared to our study. In a study from China, the percentages of physicians and nurses who experienced moderate to severe anxiety were 11.98% and 14.90%, respectively (Que *et al.*, 2020). The high prevalence of stress and generalized anxiety disorder among HCWs might be explained by a host of variables, including uncertainty surrounding the present and future course of the COVID-19 pandemic. In the absence of a vaccine and cure for COVID-19, the pandemic will remain, unfortunately, a source of stress with psychological morbidity for HCWs. We found no correlation between age and GAD. Although we have little research on the role of age in GAD experience among HCWs during the current pandemic, a study of HCWs in Iraq (Abdulah & Musa, 2020) and Italy (Rossi *et al.*, 2020) found that a higher level of GAD was associated with younger age.

The well-being of HCWs in our study, as gauged by WHO-5, was low regardless of gender and occupation. The mean score of 51 in our study is much lower than the mean score of 62 obtained from a Danish study during COVID-19 (Sønderskov *et al.*, 2020). This an expected outcome considering the high levels of stress and anxiety experienced by the participants.

The high levels of stress and anxiety and the low level of wellness reported here are indicative of the substantial damage inflicted upon HCWs in Oman by the current global health crisis. The health authority in Oman should conduct an in-depth mental health assessment of HCWs to identify individuals in need of immediate psychological attention.

Supporting and maintaining a healthy healthcare workforce is vital during this pandemic. Hence it is important to consider published guidance in relation to helping HCWs (Chen *et al.*, 2020). Dewey *et al.* (2020) suggest that leaders of health institutions should communicate their appreciation to frontline clinicians, monitor their wellness, and encourage them to discuss their concerns and vulnerabilities (Dewey *et al.* 2020).

Healthcare authorities should benefit from the existing approaches and mechanisms to implement and expand the use of telemedicine to protect patients and HCWs from exposure to COVID-19 (Bhaskar *et al.*, 2020). Also, with the large increase in teletherapy use during COVID-19 (Pierce *et al.*, 2020), psychiatrists and psychologists should consider developing strategies to deliver teletherapy to support and treat mental health problems among HCWs. Support and resources for teletherapy are already available from many professional organizations, including the American Psychological Association, the American Psychiatric Association, and the European Federation of Psychologists’ Associations.

## Limitations

Although this is one of the earliest studies to examine the mental health of HCWs in the Arab world the study has several limitations. As a cross-sectional study, we cannot draw conclusions about causality. There is a need for well-designed longitudinal studies that track frontline HCWs’ mental health over an extended period, as well as for further comparative studies in other countries. As the data was collected online the results may be influenced by selection bias. Future studies on COVID-19 should compare the experience of HCWs working on the frontline to those who have minimal or no contact with COVID-19 patients.

## Conclusion

To our knowledge, this is the first study to report on the mental health of HCWs managing COVID-19 in the Arab world. The findings of this study showed that HCWs are particularly vulnerable during the current global health crisis. Specifically, we found that HCWs from Oman experienced high levels of stress and anxiety and low levels of well-being. The outcomes of the present study are consistent with a growing body of literature demonstrating the psychological impact of COVID-19 on physicians and nurses worldwide. We urge healthcare leaders internationally to expand the use of telemedicine and set up mental health support systems for HCWs, especially those who work closely with COVID-19 patients.
